# Thermal-Related Stress–Strain Behavior of Alkali Activated Slag Concretes under Compression

**DOI:** 10.3390/ma16093470

**Published:** 2023-04-29

**Authors:** Miao Zhang, Qianmin Ma, Yang Chen, Zhuo Liu, Haoxue Zhou

**Affiliations:** 1 Faculty of Civil Engineering and Mechanics, Kunming University of Science and Technology, Kunming 650504, China; 2 Yunnan Key Laboratory of Disaster Reduction in Civil Engineering, Kunming 650500, China

**Keywords:** alkali-activated slag concrete, high temperature, stress–strain behavior

## Abstract

In this paper, the thermal-related stress–strain behavior of alkali-activated slag (AAS) concretes, with different alkali concentrations and moduli, was studied under compression. After exposure to high temperatures (200 °C, 400 °C, 600 °C, 800 °C, and 1000 °C), a compression test was carried out on the specimens. The stress–strain relationship, axial compressive strength, and elastic modulus were expressed using both a displacement extensometer and the digital image correlation (DIC) technique. It was mainly determined that: (1) With the increase in temperature, the stress–strain curves of the AAS concretes tended to be flattened, indicating reductions in both axial compressive strength and elastic modulus. After 1000 °C, only 2.5–3.7% axial compressive strength and 1.4–3.9% elastic modulus remained, respectively. (2) The DIC technique was used for thermal strain measurements of the AAS concrete. Compared to the traditional extensometer, DIC yielded a small error of 4.5% and 7.2% for axial compressive strength and elastic modulus measurements, respectively. The strain cloud chart obtained from DIC was helpful for monitoring the damage process of the specimens. The findings of this paper refined scientific systems of AAS concrete under thermal action, and also provided a newly non-contact approach for thermal strain measurements of AAS concrete under compression.

## 1. Introduction

Alkali-activated slag (AAS) is made by activating granulated blast furnace slag (GGBS) with an alkaline activator (sodium silicate, etc.) [1]. As it does not contain cement clinker and uses fully solid waste resources, AAS has been treated as an environmentally friendly and resource-saving green sustainable building material [2]. Additionally, because of its advantages of low carbon dioxide emissions, high strength, low hydration heat, and superior durability, AAS has great potential to be used as a cementitious material to manufacture structural concrete [3]. 

Fires have frequently occurred in buildings in recent years. In this context, as a potential cementitious material with which to construct buildings, the fire (high temperature)-related performance, and mechanical properties in particular, of AAS should be fully understood before its extensive application. Table 1 summarizes studies on the mechanical properties of AAS after exposure to high temperatures [4,5,6,7,8,9,10,11,12,13,14,15,16,17,18,19,20,21,22,23,24]. From the table, it can be seen that a certain number of studies have already been carried out on this topic. However, most studies paid much more attention to AAS paste and mortar specimens, while studies on AAS concrete specimens are still limited. Compared to concrete, which is key at a structural level, paste and mortar are less significant in guiding engineering practice. Furthermore, in the limited studies related to concrete specimens, normal AAS concrete has not been fully considered (as specified in previous remarks), resulting in the conclusion that these studies could be less representative and less universally applicable. Although normal AAS concrete was considered in one such study [22], its basic behavior under compression was not explored. Therefore, in order to refine the scientific system of AAS and enlarge its application in structural engineering, it is necessary to carry out a fundamental investigation on the thermal mechanical properties of AAS concrete under compression.

Compared to a compressive strength test, which is usually applied to evaluate the mechanical properties of concrete after high-temperature treatments, the stress–strain behavior could reflect its bearing capacity and deformation more comprehensively. Researchers have discussed the thermal stress–strain behavior of normal concrete [25,26,27], fiber-reinforced concrete [28,29], recycled aggregate concrete [30,31,32], reactive powder concrete [33], lightweight aggregate concrete [34], and high-strength concrete [35]. Yang et al. [20] innovatively studied the thermal stress–strain behavior of AAS ultra-high strength concrete. However, (1) the current guidance on ordinary AAS concrete is still limited, and DIC technology can better observe its thermal stress–strain behavior; (2) the mechanical properties of the AAS concrete system after exposure to high temperature are not perfect, and the application of non-contact measurements in AAS concrete deformation measurement should be expanded. Therefore, the thermal stress–strain behavior of normal AAS concrete under axial compression is studied in this paper. On the basis of traditional strain measurement using a displacement extensometer, digital image correlation (DIC) technology is also applied in this paper in order to measure the thermal strain of AAS concrete, due to its success in normal concrete [36], with an attempt to provide a non-contact approach for such measurements of AAS concrete. 

## 2. Experiment

In this paper, after exposure to high temperatures, uniaxial compression was carried out on AAS concretes (sodium silicate solution was used as an activator) with different alkali concentrations (Na_2_O% of slag, 4, 6, 8) and moduli (Ms, 1.0, 1.5, 2.0). The stress–strain relationship, axial compressive strength, and the elastic modulus of the concretes was studied. 

### 2.1. Materials

**Figure 1:**
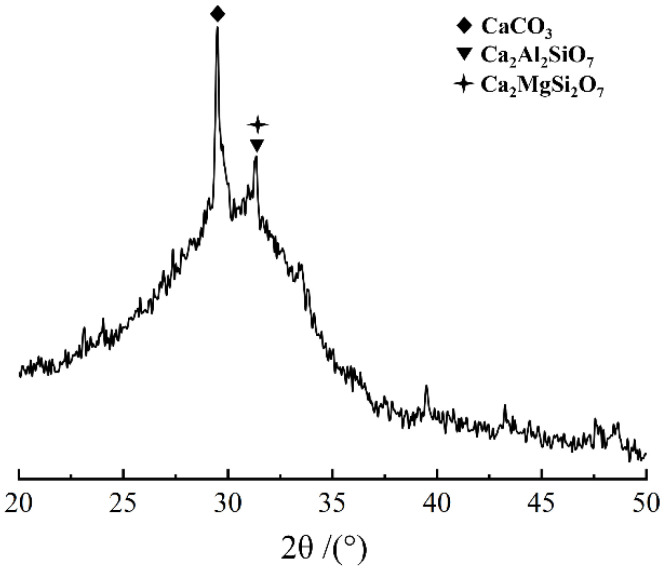
XRD pattern of slag.

Slag from Qujing, Yunnan province, China, was used to manufacture AAS concrete. The activity index of the slag was 83% at 28 days. Its chemical compositions and X-ray diffraction (XRD) pattern are shown in Table 2 and Figure 1, respectively. From the XRD pattern it can be seen that the slag is mainly amorphous, thought it contains a small amount of crystalline minerals such as calcite, gehlenite and akermanite. Industrial sodium silicate solution (water glass, WG) produced locally was used as alkali activator; its contents of SiO_2_% and Na_2_O% were 22.22% and 7.51%, respectively. The original Ms of the WG is 3.08, and therefore, NaOH (with purity no less than 96%) produced by Fuchen chemical reagent Co., Ltd. in Tianjin, China, was used to adjust the Ms of the WG to the values required. Manufactured sand with a modulus of 2.81 was used as fine aggregates. Before mixing, the sand was sieved sufficiently to remove any stone powder that might exist. Crushed limestone with a continuous gradation in a range of 5–25 mm was used as coarse aggregate. Tap water was used for mixing and curing. 

### 2.2. Specimens Preparation

AAS concretes with Na_2_O% of 4, 6 and 8, and Ms of sodium silicate solution of 1.0, 1.5 and 2.0 were produced. For all the mixes, the binder content, water binder ratio (W/B), and sand ratio were kept same at 400 kg/m^3^, 0.45 and 40%, respectively. Binder content was a sum of slag content, NaOH content and solid content in WG. Water content in WG was considered in the calculation of W/B. The mix proportions of the concretes are given in Table 3. The size of the concrete specimens was 100 mm × 100 mm × 300 mm. After mixing, the concrete mixture was poured in the mold in two layers. After each layer casting, the mixture in the mold was placed on a vibration table and vibrated until the mixture was compacted. After that, the mixture was cured in the mold under a condition with temperature of 20 ± 2 °C and relative humidity higher than 95% for 24 h. Later, the specimens were demolded and cured under the same conditions for 90 days. Three parallel specimens were prepared for each mix at each temperature condition, and the results to be reported are the average of the three measurements.

### 2.3. Heating Regime

**Figure 2:**
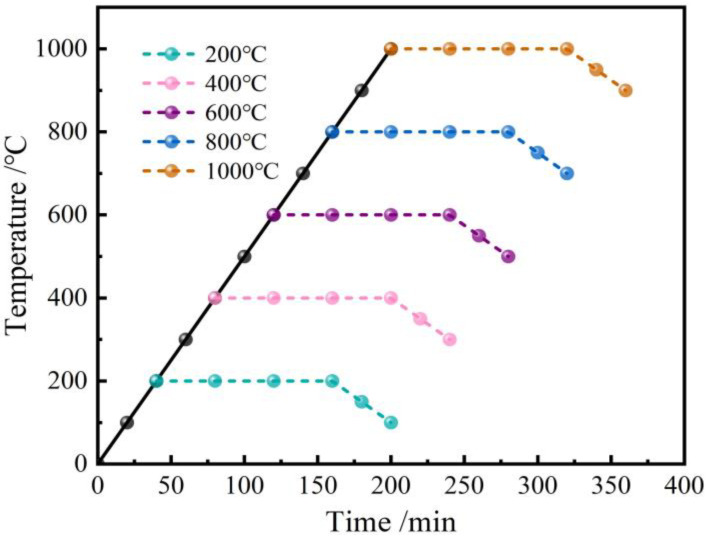
Heating regime.

A muffle furnace was used to heat the concrete specimens to 200 °C, 400 °C, 600 °C, 800 °C, and 1000 °C, respectively, with a heating velocity of 5 °C/min. After maintaining 2 h at the target temperatures, the power of the furnace was shut down and the specimens were cooled with the furnace to room temperature. The regime described above is shown in Figure 2.

### 2.4. Axial Compression Testing

#### 2.4.1. Displacement Extensometer Measurement

**Figure 3:**
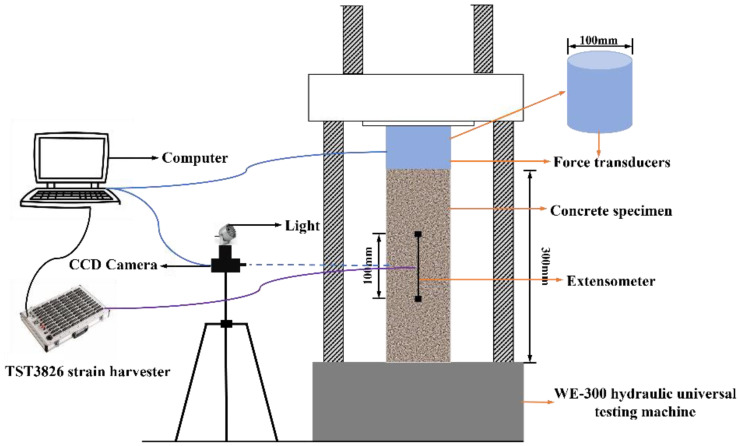
Schematic diagram of the experimental setup.

An axial compression test was carried out on the concrete specimens at room temperature and after high-temperature exposures according to the Chinese standard GB/T50081-2019 [37]. The experimental setup is shown schematically in Figure 3. A WE-300 hydraulic universal testing system was used to apply an axial load to the specimens at a uniform rate of 0.5 MPa/s~0.8 MPa/s until the specimen was damaged. A computer was used to connect both a force sensor and a testing system TST3826 for strain measurement. Displacement extensometers were installed on any two opposite sides of the specimen to detect its deformation under the compression, with a measuring distance of 100 mm.

Axial compressive strength $f_{c}$ (MPa) of the concrete specimens was calculated according to Equation (1):

(1) $$f_{c}=\frac{F}{A}$$

The elastic modulus E (GPa) of the concrete specimens was calculated by using Equations (2) and (3):

(2) $$E=\frac{F_{a}-F_{0}}{A}\times \frac{L}{∆n}$$

(3) $$∆n=\varepsilon_{a}-\varepsilon_{0}$$

where

*F* is the maximum force the specimens could withstand, kN; 

*F*_0_ is the initial force when reference stress is 0.5 MPa, kN; 

*F_a_* is the force when stress is 1/3 of the $f_{c}$, kN;

*A* is the loading area, 100 mm × 100 mm;

*L* is the measure distance, 100 mm;

*ε*_0_ is the deformation value recorded in the last 30 s while maintaining *F*_0_ for 60 s, mm; 

*ε_a_* is the deformation value recorded in the last 30 s while maintaining *F_a_* for 60 s, mm.

#### 2.4.2. DIC Detection

Simultaneously, DIC technology was also used to test and analyze the stress–strain behavior of the concretes under axial compression, and the results were compared to the ones obtained from displacement extensometers. 

**Figure 4:**
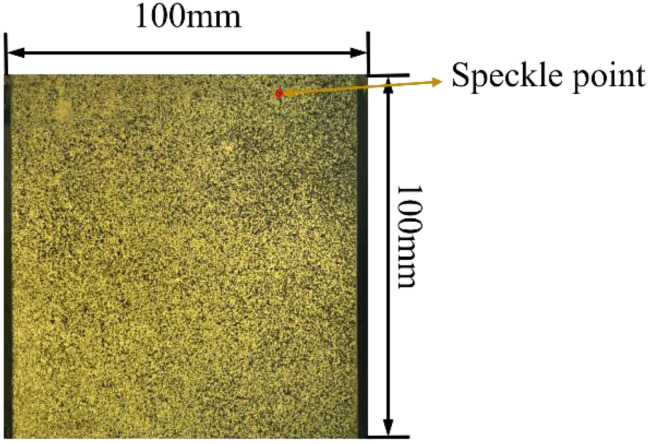
Artificial speckle on the surface of the concrete specimens.

**Figure 5:**
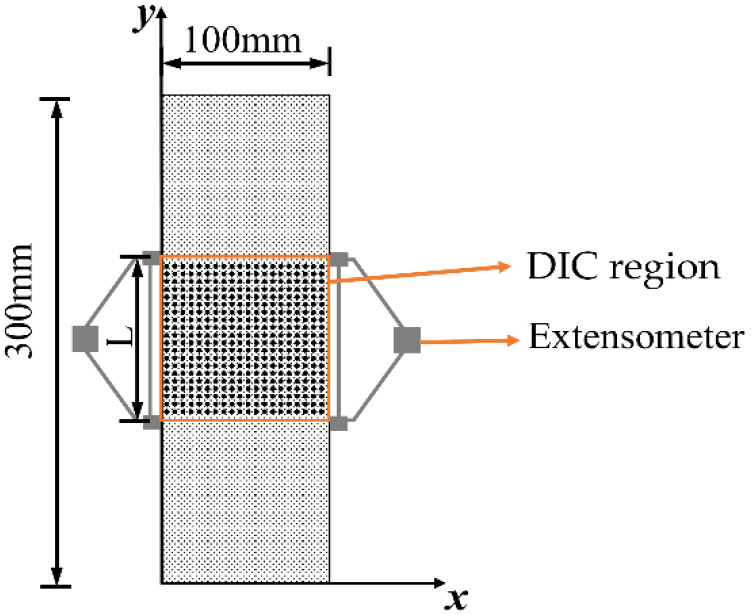
Schematic diagram of inspection points.

Before the testing, white paint was sprayed onto the surface of the two sides of the specimens with no displacement extensometers, followed by black paint spraying, to artificially produce random speckles (see Figure 4) for deformation tracking. As shown in Figure 3, a charge coupled device (CCD) camera was placed facing the testing surface to collect its images during the compression with a frequency of one image per second. Application of light could improve the quality of the images collected. After that, VIC-2D 2009 software was applied to process the images, while only the region covered by the extensometers was focused on for the purpose of comparison. The region was divided equally into 20 points along with x and y directions, respectively; therefore, a total of 400 points were obtained in the region (see Figure 5). The displacement of each point was then calculated using the VIC-2D 2009 softw are and the results were converted into numbers with the assistance of MATLAB. The displacement values of the 20 points at the top and at the bottom, respectively, were used to calculate the strain $\varepsilon_{r}$ of the region using Equation (4).

(4) $$\varepsilon_{r}=\frac{\left|\overset{-}{D_{t}}-\overset{-}{D_{b}}\right|}{L}$$

where 

$\overset{-}{D_{t}}$ is the average displacement of the 20 points at the top of the region, mm;

$\overset{-}{D_{b}}$ is the average displacement of the 20 points at the bottom of the region, mm;

L is the distance between the top and the bottom, 100 mm.

Furthermore, VIC-2D was also used to calculate the strain field of the region, where the step size and sub-region were assigned to 5 pixels and 151 pixels × 151 pixels, respectively. 

## 3. Results and Discussion

### 3.1. Stress–Strain Relationship

**Figure 6:**
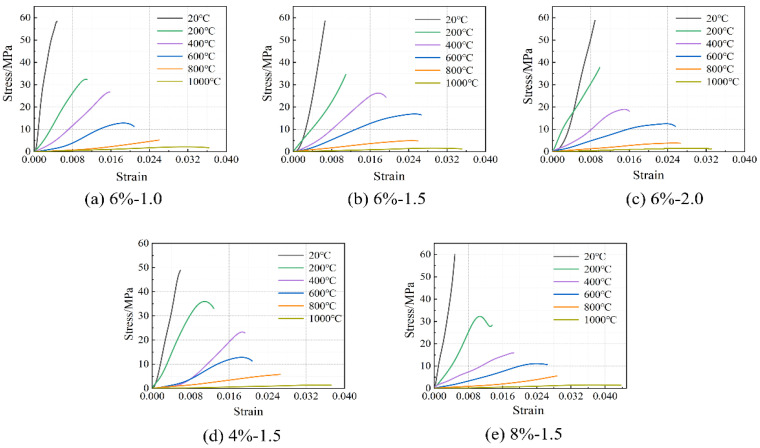
Stress–strain relationship of the concrete specimens at room temperature and after exposure to high temperatures.

**Figure 7:**
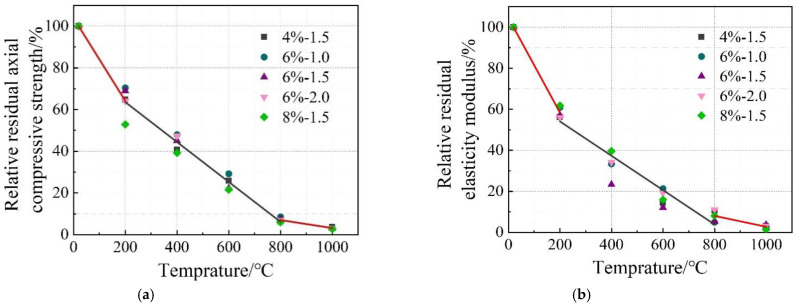
Relative residual axial compressive strength and relative residual elastic modulus of the concrete. (**a**) Axial compressive strength. (**b**) Elastic modulus.

The stress–strain relationship of the concrete specimens at room temperature and after exposure to high temperatures is shown in Figure 6. The stress peak (σ_p_, axial compressive strength), gradient of the curve (elastic modulus, E), and the strain corresponding to the stress peak (ε) are summarized in Table 4. Generally, with the increase in temperature, the stress–strain curves tend to be flattened, indicating reductions in both the stress peak (axial compressive strength) and the gradient of the curve (elastic modulus), as well as an increase in the strain corresponding to the stress peak. After reaching a temperature of 800 °C, there was nearly no axial compressive strength and elastic modulus remaining. After reaching 1000 °C, only 2.5–3.7% axial compressive strength and 1.4–3.9% elastic modulus remained, respectively. With the increase in temperature, as a result of continuous decomposition of hydrates [12], formation of porous structure [38], and inconsistent deformation between matrix and aggregates [11], the stress–strain relationship described above is quite similar to that of conventional concrete [38]. Furthermore, it is in accordance with the results reported by Yang et al. [20]. However, AAS ultra-high-strength concretes were considered. From Figure 7, it can be seen that the reductions in both axial compressive strength and elastic modulus with temperature could be divided generally into three stages, as described in Equations (5) and (6), respectively.

(5) $$\frac{f_{a}}{f_{a0}}=\left\{\begin{array}{ll} 103.978-0.199T\;20\leq T\leq 200 & R^{2}=0.9442\\ 82.738-0.096T\;200\leq T\leq 800 & R^{2}=0.9671\\ 22.299-0.019T\;800\leq T\leq 1000 & R^{2}=0.8855 \end{array}\right.$$

where

*f_a_* is the axial compressive strength of the concrete after exposure to high temperatures, MPa; *f_a_*_0_ is the axial compressive strength of the concrete at room temperature, MPa;

*T* is the temperature, °C.

(6) $$\frac{E}{E_{0}}=\left\{\begin{array}{ll} 104.622-0.231T\;20\leq T\leq 200 & R^{2}=0.9931\\ 70.833-0.084T\;200\leq T\leq 800 & R^{2}=0.9187\\ 29.170-0.010T\;800\leq T\leq 1000 & R^{2}=0.6851 \end{array}\right.$$

where 

*E* is the elastic modulus of the concrete after exposure to high temperatures, GPa;

*E*_0_ is the elastic modulus of the concrete at room temperature, GPa;

*T* is the temperature, °C.

**Table 4:** Axial compressive strength, elastic modulus, and strain corresponding to the stress peak.

		T (°C)					
		20	200	400	600	800	1000
6%-1.0	σ_p_ (MPa)	58.4 (±3.3)	32.4 (±4.0)	26.8 (±2.0)	12.8 (±1.4)	5.2 (±0.9)	2.2 (±0.6)
	E (GPa)	44.0 (±1.8)	22.1 (±3.3)	12.7 (±1.3)	6.6 (±0.6)	2.6 (±0.3)	1.0 (±0.2)
	ε (×10^−3^)	4.8 (±0.9)	11.1 (±1.1)	15.7 (±1.4)	20.9 (±5.2)	26.0 (±2.7)	36.2 (±3.4)
6%-1.5	σ_p_ (MPa)	58.6 (±3.0)	34.5 (±0.7)	24.2 (±3.1)	16.5 (±2.4)	4.9 (±0.7)	1.6 (±0.1)
	E (GPa)	43.7 (±2.5)	26.3 (±2.6)	11.1 (±2.5)	5.7 (±0.9)	2.2 (±0.2)	1.7 (±0.2)
	ε (×10^−3^)	6.6 (±1.1)	10.9 (±0.9)	19.3 (±1.8)	26.6 (±2.7)	25.7 (±3.8)	35.1 (±5.3)
6%-2.0	σ_p_ (MPa)	58.8 (±1.9)	37.7 (±2.0)	19.0 (±3.7)	12.5 (±0.8)	3.9 (±0.6)	1.5 (±0.1)
	E (GPa)	42.7 (±0.6)	25.5 (±2.8)	11.9 (±1.4)	5.3 (±0.6)	2.1 (±0.5)	1.4 (±0.3)
	ε (×10^−3^)	8.9 (±0.8)	9.9 (±1.5)	16.0 (±3.2)	25.6 (±3.4)	26.6 (±5.0)	33.4 (±4.1)
4%-1.5	σ_p_ (MPa)	48.9 (±2.4)	36.0 (±2.3)	23.4 (±1.3)	12.8 (±2.9)	5.8 (±1.2)	1.4 (±0.6)
	E (GPa)	45.6 (±1.6)	24.1 (±2.5)	11.6 (±0.8)	6.4 (±3.0)	1.7 (±0.7)	1.4 (±0.3)
	ε (×10^−3^)	6.3 (±1.4)	11.9 (±1.1)	19.6 (±2.3)	20.6 (±2.2)	27.9 (±4.8)	36.1 (±3.4)
8%-1.5	σ_p_ (MPa)	60.0 (±5.8)	32.3 (±3.9)	15.9 (±2.0)	11.1 (±1.9)	5.5 (±1.1)	1.5 (±0.4
	E (GPa)	43.6 (±3.0)	25.9 (±3.5)	9.1 (±2.3)	2.8 (±0.6)	1.6 (±0.5)	0.6 (±0.1)
	ε (×10^−3^)	4.8 (±1.2)	13.5 (±0.9)	18.6 (±1.4)	24.5 (±2.5)	28.7 (±3.2)	42.6 (±5.4)

### 3.2. Effect of Na_2_O% and Ms

**Figure 8:**
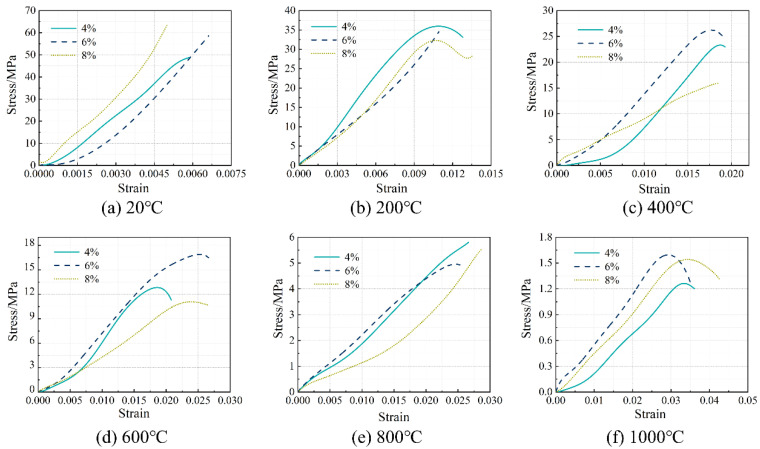
Effect of Na_2_O% on the stress–strain relationship of the concrete specimens.

**Figure 9:**
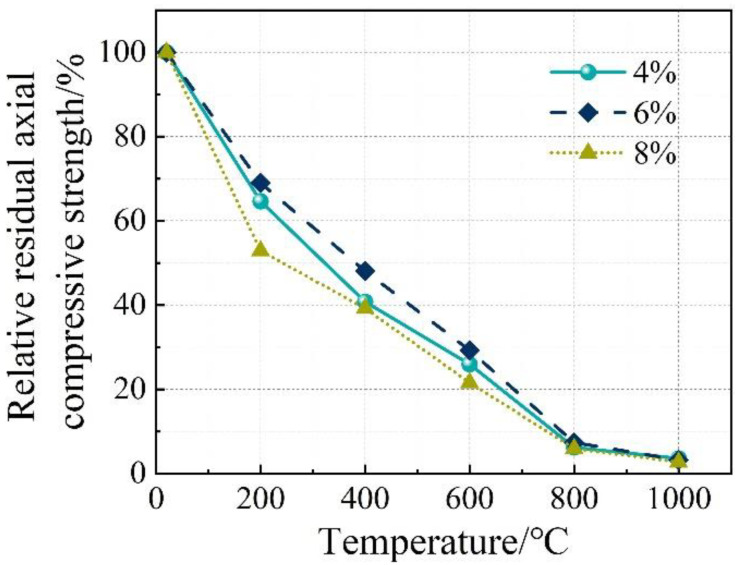
Effect of Na_2_O% on the relative residual axial compressive strength of the concrete specimens.

**Figure 10:**
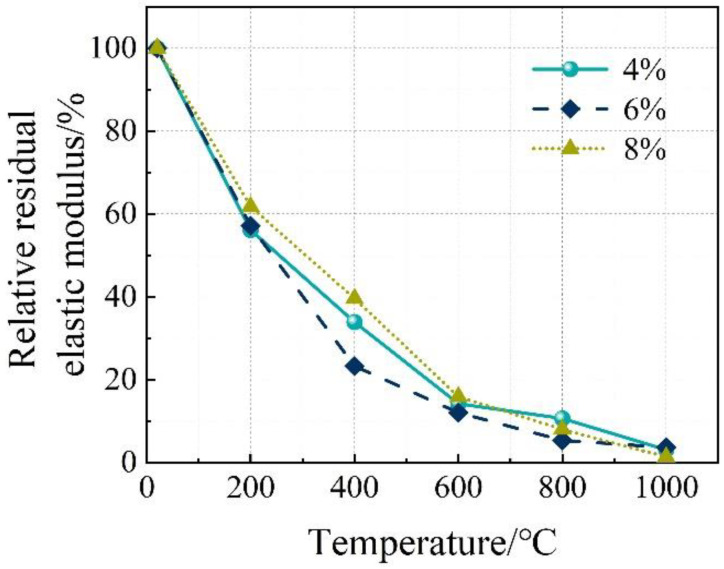
Effect of Na_2_O% on the relative residual elastic modulus of the concrete specimens.

**Figure 11:**
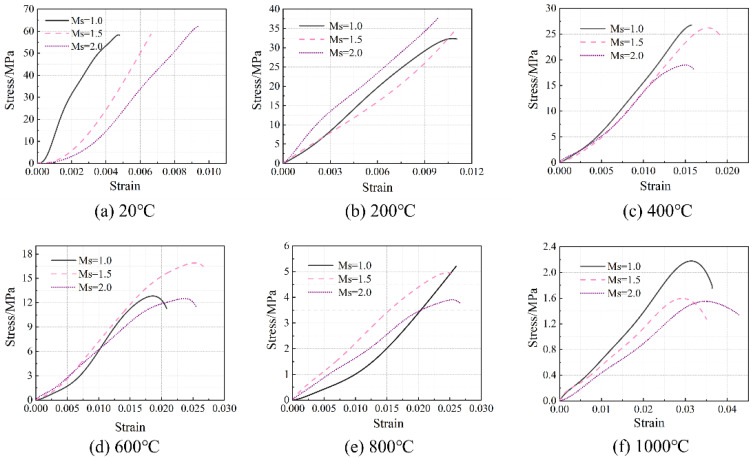
Effect of Ms on stress–strain relationship of the concrete specimens.

**Figure 12:**
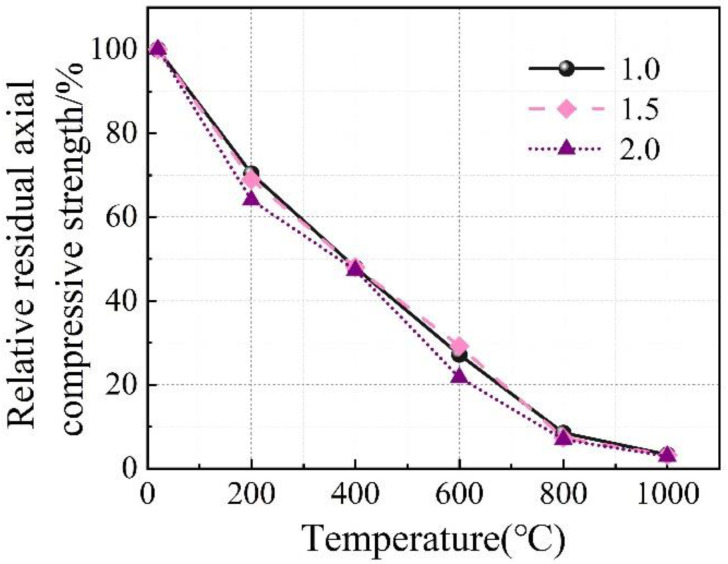
Effect of Ms on the relative residual axial compressive strength of the concrete specimens.

**Figure 13:**
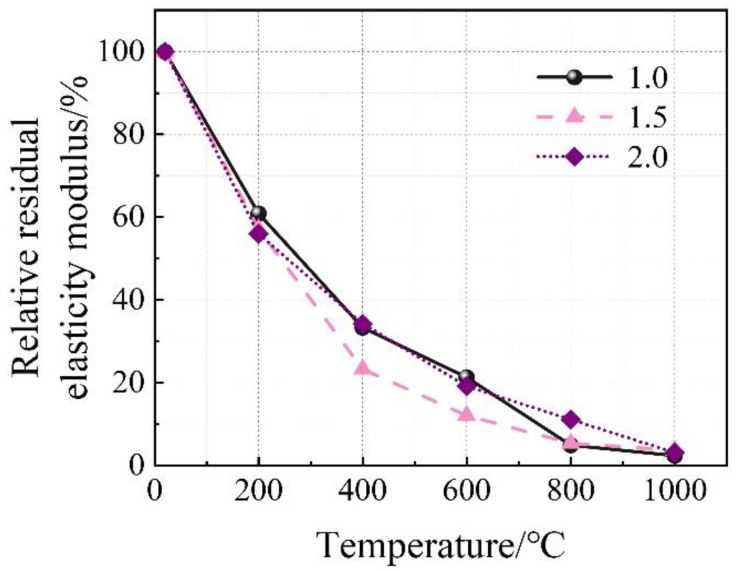
Effect of Ms on the relative residual elastic modulus of the concrete specimens.

The influence of Na_2_O% on the stress–strain relationship of the concrete specimens at room temperature and after exposure to high temperatures is shown in Figure 8, where Ms was kept at 1.5. Furthermore, such an influence on the axial compressive strength and elastic modulus is illustrated in Figure 9 and Figure 10, respectively. Similarly, the influence of Ms on the performance mentioned above is exhibited in Figure 11, Figure 12 and Figure 13 where Na_2_O% was kept at 6%. From Figure 9, it can be seen that before 600 °C, Na_2_O% of 6% could ensure a relatively higher residual axial compressive strength. This is in agreement with the results reported by Nasr et al. [19]. The reason for this could be because such Na_2_O% could provide AAS with sufficient hydration and then a dense matrix structure [39] to resist thermal damage. After reaching 600 °C, the influence of Na_2_O% on the residual axial compressive strength of the concrete specimens was not significant. However, unlike axial compressive strength, the concrete specimen with Na_2_O% of 6% did not exhibit much advantage when the residual elastic modulus was considered before 600 °C, as shown in Figure 10. Similarly, Na_2_O% had no significant influence on the elastic modulus of the concrete after 600 °C. When Ms of 2.0 was applied, the concrete specimen always yielded a relatively lower residual axial compressive strength as a result of the less hydrated matrix [40]. Meanwhile, when the residual elastic modulus was considered, such mixes performed well to a certain extent. 

### 3.3. Comparison between Extensometer and DIC

**Figure 14:**
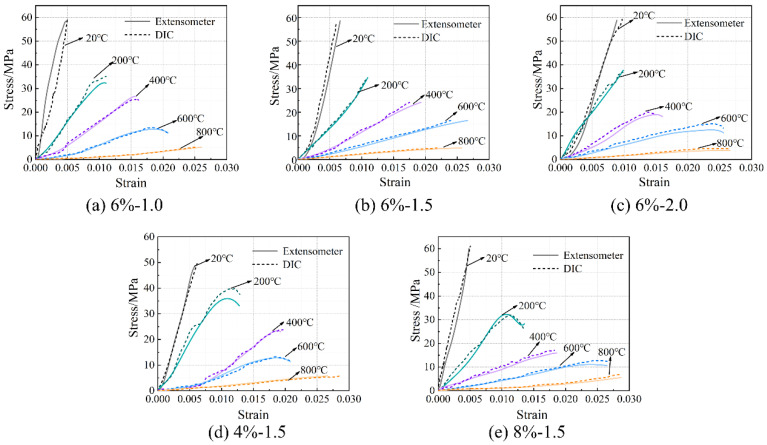
Comparison of stress–strain relationship obtained using an extensometer and DIC.

The thermal stress–strain relationship obtained using DIC technology is shown in Figure 14 and compared to that obtained using an extensometer to verify the feasibility of DIC technology for thermal strain measurement of the AAS concrete under axial compression. As a temperature of 1000 °C resulted in cracking on the surface of the specimens, inducing damage in the form of speckles, the stress–strain relationship of the concrete specimens after 1000 °C is missing in the figure. From the figure, it can be seen that the stress–strain curves of the concrete specimens obtained using the DIC technique are in good agreement with those measured using an extensometer. However, it should be noted that in general, the curves obtained using DIC are not as smooth as the ones obtained using an extensometer. This is a result of incomplete strain measurement, which could be caused by (1) the specimens not deforming uniformly under loading, and (2) the size and shape of the speckles on the surface of the specimens changing during loading. The axial compressive strength and elastic modulus measured by DIC are given in Table 5, as is the error between the values measured by using DIC and extensometer. From the table, it can be seen that the error of axial compressive strength is in a range of [0.1%, 14.6%] with an average of 4.5%. Such value for elastic modulus is in a range of [2.0%, 28.9%] with an average of 7.2%. Both the error values are acceptable for engineering practice, confirming the feasibility of the DIC technique and providing a newly non-contact approach for thermal strain measurement of the AAS concrete under axial compression. Different testing objects (speckles for DIC and fixed points for extensometer) and testing environments (lights for DIC) could have caused the errors. 

### 3.4. Strain Cloud Chart

**Figure 15:**
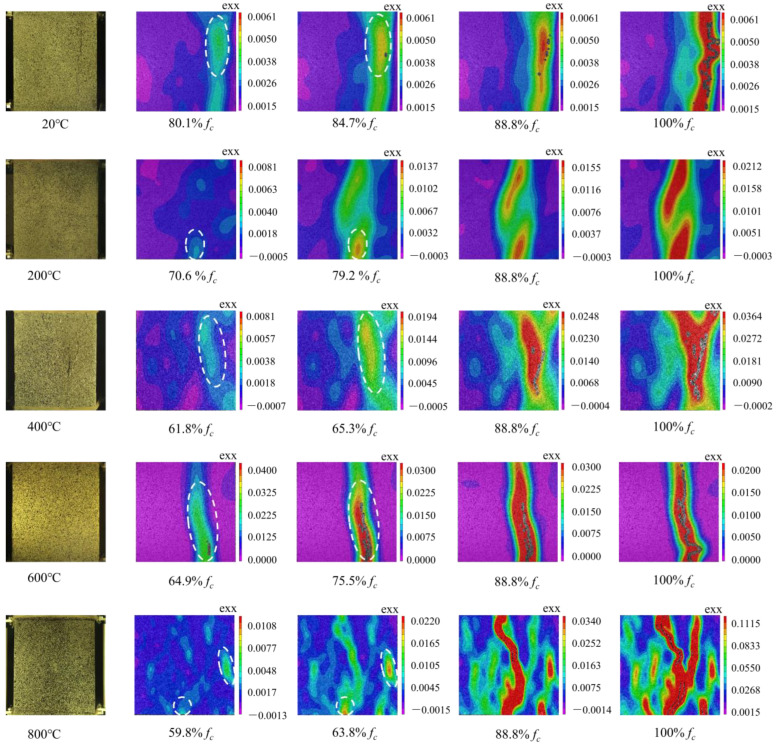
Strain cloud chart.

Since the strain value in the cracked area is greater than that in the non-cracked area, the cracks could be easily identified according to the strain on the concrete surface. After high-temperature exposure, the damage process of the concrete specimens under axial compression was monitored using the DIC technique in situ, and the result (6%-2.0 as an example) is shown in Figure 15. In the figure, the region whose color is strongly different from the background is considered to be clear strain caused by cracking. Color deepening indicates an increase in the strain caused by more severe cracking. From the figure, it can be seen that in general, the stress corresponding to the first cracking of the specimens reduced with the increase in temperature, as a result of thermal damage. After the initial cracking, with the further increase in stress, more severe cracking occurred on the specimens. When cracks went through the section of the specimens, the specimens were completely damaged. 

## 4. Conclusions

This paper focuses on the axial compression performance of AAS concrete exposed to high temperatures. In particularly, the effect of high temperature, different alkali concentration, and modulus on the residual axial compressive strength, elastic, modulus, and stress–strain behavior have been analyzed in detail. Based on the experimental program used in this paper, the following conclusions could be drawn:

(1) Generally, with the increase in temperature, stress–strain curves of the AAS concrete specimens tend to be flattened, indicating reductions in both stress peak (axial compressive strength) and gradient of the curve (elastic modulus), as well as an increase in the strain corresponding to the stress peak. After 1000 °C, only 2.5–3.7% axial compressive strength and 1.4–3.9% elastic modulus remained, respectively.
(2) Before 600 °C, Na_2_O% of 6% could ensure a relatively higher residual axial compressive strength. However, the concrete specimen with Na_2_O% of 6% did not exhibit much advantage when residual elastic modulus was considered. After reaching 600 °C, the influence of Na_2_O% on both axial compressive strength and elastic modulus was not significant. When Ms of 2.0 was applied, the concrete specimen always yielded a relatively lower residual axial compressive strength. Meanwhile, when residual elastic modulus was considered, such mixes performed well to a certain extent.
(3) The DIC technique is feasible for thermal strain measurement of the AAS concrete. Compared to a traditional extensometer, DIC has yielded a small error of 4.5% and 7.2%, respectively, for axial compressive strength and elastic modulus measurement. The strain cloud chart obtained using DIC has confirmed that in general, the stress corresponding to the first cracking of the specimens reduced with the increase in temperature, as a result of thermal damage. After the initial cracking, with the further increase in stress, more severe cracking occurred on the specimens. When cracks went through the section of the specimens, the specimens were completely damaged.

Overall, the findings of this study have refined the scientific system of AAS concrete under thermal action, and also have provided a newly non-contact approach for thermal strain measurement of AAS concrete under compression.

## Figures and Tables

**Table 1:** Summary of studies on the thermal mechanical properties of AAS.

Ref.	Type of Specimen	Temperature (°C)	Type of Alkali Activator	Modulus *	Alkali Concentration (Na_2_O% of Precursor)	Mechanical Properties Tested	Remarks
[4]	Paste	200, 400, 600, 800, 1000	Sodium silicate solution	1.7	3.5, 5.5, 6.5, 10.5	Compressive strength	
[5]	Paste	600, 800, 1000, 1200	Sodium silicate solution	1.0	5	Compressive strength	
[6]	Paste	200, 500, 800	Sodium silicate solution	0.50, 0.75, 1.00	7–10	Compressive strength	
[7]	Paste	200, 400, 600, 800, 1000, 1200	Sodium silicate solution	1.95	5	Compressive strength, flexural strength	
[8]	Paste	200, 400, 600, 800	Sodium sulfate	--	1, 3	Compressive strength	
[9]	Mortar	200, 400, 600, 800, 1000	Sodium silicate solution	1.0	4	Compressive strength, flexural strength, tensile strength	
[10]	Mortar	200, 400, 600, 800	Sodium silicate solution	2.4	4	Compressive strength	
[11]	Mortar	200, 400, 600, 800, 1000, 1200	Sodium silicate solution	1.0	4, 6, 8, 10	Compressive strength, flexural strength, tensile strength	
[12]	Mortar	400, 600, 800	Sodium silicate solution	0.25, 0.50, 0.75, 1.00, 1.25	6	Compressive strength, flexural strength	
[13]	Mortar	200, 400, 600, 800	Sodium silicate solution	1	14	Compressive strength, flexural strength	
[14]	Mortar	300, 450, 600, 750, 900, 1050	Sodium silicate solution	0.1	2, 4, 6, 8, 10	Compressive strength, flexural strength	50% GGBS + 50% basaltic pumice
[15]	Mortar	300, 600	Sodium metasilicate	1.0	1.2	Compressive strength	
[16]	Mortar	200, 400, 600, 800	Sodium silicate solution	0.85	13.5	Compressive strength	
[17]	Mortar	200, 400, 600, 800	NaOH	--	31	Compressive strength	
[18]	Mortar	200, 400, 600, 800	Sodium silicate solution	0.6	21	Compressive strength	30% GGBS + 70% fly ash
[19]	Mortar	200, 400, 600, 800	Sodium silicate solution	2.3	2, 4, 6, 8, 10, 12	Compressive strength, flexural strength	
[20]	Concrete	200, 400, 600, 800, 1000	Combination of potassium carbonate and sodium silicate	--	N/A	Stress–strain behavior	Ultra-high-strength concrete (70% GGBS + 10% quatz powder + 20% silica fume)
[21]	Concrete	200, 400, 600, 800	Sodium silicate solution	1.3	5	Compressive strength, splitting tensile strength	Concrete with sea sand and sea water (75% GGBS + 25% fly ash, 50% GGBS + 50% fly ash)
[22]	Concrete	200, 400, 600, 800	Sodium silicate solution	0.4	23	Tensile strength	
[23]	Concrete	400, 600, 800	Combination of potassium carbonate, sodium silicate, and sodium carbonate	--	N/A	Compressive strength	Ultra-high-strength concrete ((70% GGBS + 10% quatz powder + 20% silica fume)
[24]	Concrete	200, 400, 600, 800	Sodium silicate solution	1.0	5–7	Compressive strength	Self-compacting high-performance concrete

* (1) Modulus is mole ratio between SiO_2_ and Na_2_O when sodium silicate solution was used as alkali activator. (2) N/A is an abbreviation for not available.

**Table 2:** Chemical compositions of slag (%).

CaO	SiO_2_	Al_2_O_3_	MgO	TiO_2_	SO_3_	MnO	K_2_O	Fe_2_O_3_
40.68	33.48	13.43	5.70	2.57	1.98	1.03	0.55	0.29

**Table 3:** Mix proportions of the concretes (kg/m^3^).

Specimen No. (Na_2_O%-Ms)	Slag	WG	NaOH	Coarse Aggregate	Fine Aggregate	Water
4%-1.5	344	90	9	1139	759	108
6%-1.0	336	88	18	1143	762	109
6%-1.5	329	129	13	1164	776	80
6%-2.0	321	168	9	1185	790	53
8%-1.5	314	164	16	1188	792	55

**Table 5:** Comparison of axial compressive strength and elastic modulus measured using DIC and an extensometer.

			T (°C)				
			20	200	400	600	800
6%-1.0	σ_p_ (MPa)	DIC	59.3	35.1	25.9	13.5	5.3
		Extensometer	58.4	32.4	26.8	12.8	5.2
		Error (%)	1.51	7.62	3.35	4.95	1.31
	E (GPa)	DIC	45.7	23.4	13.4	6.9	2.7
		Extensometer	44.0	22.1	12.7	6.6	2.6
		Error (%)	3.75	5.74	5.02	5.13	4.00
6%-1.5	σ_p_ (MPa)	DIC	57.1	34.3	24.2	16.5	4.9
		Extensometer	58.6	34.5	24.2	16.5	4.9
		Error (%)	2.645	0.82	0.13	0.12	0.24
	E (GPa)	DIC	46.4	25.3	12.0	6.9	2.3
		Extensometer	43.7	26.3	11.1	5.7	2.2
		Error (%)	5.85	3.80	7.98	17.82	5.49
6%-2.0	σ_p_ (MPa)	DIC	59.3	37.0	20.3	15.1	4.6
		Extensometer	58.8	37.7	19.0	12.5	3.9
		Error (%)	0.70	2.04	6.35	14.58	13.95
	E (GPa)	DIC	45.4	26.6	12.6	5.8	2.0
		Extensometer	42.7	25.5	11.9	5.3	2.1
		Error (%)	5.94	4.29	5.07	8.81	3.99
4%-1.5	σ_p_ (MPa)	DIC	49.7	39.9	23.7	13.3	5.8
		Extensometer	48.9	36.0	23.4	12.8	5.8
		Error (%)	1.78	9.85	1.62	3.43	0.28
	E (GPa)	DIC	46.8	26.6	11.4	4.1	1.8
		Extensometer	45.6	24.1	11.6	6.4	1.7
		Error (%)	2.64	9.60	2.03	3.51	5.65
8%-1.5	σ_p_ (MPa)	DIC	61.1	32.1	17.1	12.8	6.9
		Extensometer	60.1	32.3	15.9	11.1	5.5
		Error (%)	1.66	0.73	6.63	13.42	13.82
	E (GPa)	DIC	45.8	30.7	10.0	3.9	1.7
		Extensometer	43.6	25.9	9.1	2.8	1.6
		Error (%)	4.73	15.85	9.28	28.92	5.79

## Data Availability

Not applicable.
